# Deep learning models for predicting RNA degradation via dual crowdsourcing

**DOI:** 10.1038/s42256-022-00571-8

**Published:** 2022-12-14

**Authors:** Hannah K. Wayment-Steele, Wipapat Kladwang, Andrew M. Watkins, Do Soon Kim, Bojan Tunguz, Walter Reade, Maggie Demkin, Jonathan Romano, Roger Wellington-Oguri, John J. Nicol, Jiayang Gao, Kazuki Onodera, Kazuki Fujikawa, Hanfei Mao, Gilles Vandewiele, Michele Tinti, Bram Steenwinckel, Takuya Ito, Taiga Noumi, Shujun He, Keiichiro Ishi, Youhan Lee, Fatih Öztürk, King Yuen Chiu, Emin Öztürk, Karim Amer, Mohamed Fares, Eterna Participants, Rhiju Das

**Affiliations:** 1 Department of Chemistry, Stanford University, Stanford, CA USA; 2 Eterna Massive Open Laboratory, Stanford, CA USA; 3 Department of Biochemistry, Stanford University, Stanford, CA USA; 4 Prescient Design, Genentech, San Francisco, CA USA; 5 NVIDIA Corporation, Santa Clara, CA USA; 6 Kaggle, San Francisco, CA USA; 7 Department of Computer Science and Engineering, State University of New York at Buffalo, Buffalo, NY USA; 8 High-flyer AI, Hangzhou, Zhejiang China; 9 NVIDIA Corporation, Minato-ku, Tokyo, Japan; 10 DeNA, Shibuya-ku, Tokyo, Japan; 11 Yanfu Investments, Shanghai, China; 12 IDLab, Ghent University, Technologiepark-Zwijnaarde, Gent, Belgium; 13 The Wellcome Centre for Anti-Infectives Research, College of Life Sciences, University of Dundee, Dundee, UK; 14 Universal Knowledge Inc., Tokyo, Japan; 15 Keyence Corporation, 1-3-14, Higashi-Nakajima, Higashi-Yodogawa-ku, Osaka, Japan; 16 Department of Chemical Engineering, Texas A&M University, College Station, TX USA; 17 Rist Inc., Shimogyo-ku, Kyoto, Japan; 18 Korea Atomic Energy Research Institute, Daejeon, Republic of Korea; 19 Kakao Brain Corp, Seongnam, Gyeonggi-do Republic of Korea; 20 H2O, Istanbul, Turkey; 21 Clover Health, Hong Kong, P. R. China; 22 Afiniti, Istanbul, Turkey; 23 Center for Informatics Science, Nile University, Sheikh Zayed, Giza, Egypt; 24 National Research Centre, Dokki, Cairo, Egypt; 25 Howard Hughes Medical Institute, Stanford University, Stanford, CA USA

**Keywords:** RNA, Machine learning

## Abstract

Medicines based on messenger RNA (mRNA) hold immense potential, as evidenced by their rapid deployment as COVID-19 vaccines. However, worldwide distribution of mRNA molecules has been limited by their thermostability, which is fundamentally limited by the intrinsic instability of RNA molecules to a chemical degradation reaction called in-line hydrolysis. Predicting the degradation of an RNA molecule is a key task in designing more stable RNA-based therapeutics. Here, we describe a crowdsourced machine learning competition (‘Stanford OpenVaccine’) on Kaggle, involving single-nucleotide resolution measurements on 6,043 diverse 102–130-nucleotide RNA constructs that were themselves solicited through crowdsourcing on the RNA design platform Eterna. The entire experiment was completed in less than 6 months, and 41% of nucleotide-level predictions from the winning model were within experimental error of the ground truth measurement. Furthermore, these models generalized to blindly predicting orthogonal degradation data on much longer mRNA molecules (504–1,588 nucleotides) with improved accuracy compared with previously published models. These results indicate that such models can represent in-line hydrolysis with excellent accuracy, supporting their use for designing stabilized messenger RNAs. The integration of two crowdsourcing platforms, one for dataset creation and another for machine learning, may be fruitful for other urgent problems that demand scientific discovery on rapid timescales.

## Abstract (web summary)

Predicting RNA degradation is a fundamental task in designing RNA-based therapeutics. Two crowdsourcing platforms, Kaggle and Eterna, united to develop accurate deep learning models for RNA degradation on a timescale of 6 months.

## Main

Therapeutics based on messenger RNA (mRNA) have shown immense promise as a modular therapeutic platform, allowing potentially any protein to be delivered and translated^1,2^, as evidenced by the rapid deployment of mRNA-based vaccines against severe acute respiratory syndrome coronavirus 2 (SARS-CoV-2)^3–5^. However, the chemical instability of RNA sets a fundamental limit on the stability of RNA-based therapeutics^1,6–8^, with RNA hydrolysis specifically setting a limiting factor on stability in lipid nanoparticle (LNP)-based formulations^9,10^. Hydrolysis in LNP formulations degrades the amount of mRNA remaining during shipping and storage, and hydrolysis in vivo after vaccine injection limits the amount of resulting protein produced over time^9^. Better methods to develop thermostable RNA therapeutics would allow for increasing the equitability of their distribution, reducing their cost and possibly increasing their potency^10,11^.

An underexplored path to more shelf-stable mRNA therapeutics lies in the prospect of synonymous sequence design. A simple calculation reveals that there exist 10^633^ mRNA sequences that all code for the SARS-CoV-2 spike protein antigen. With an astronomical number of mRNA sequences available for a given therapeutic target, it is likely that some of these sequences may harbour structural features that make them more resistant to hydrolysis than first-generation mRNA vaccine formulations. Indeed, initial results have demonstrated that more stable mRNAs for model protein systems can be designed by optimizing candidate RNA sequences, scored with a model for RNA hydrolysis^12,13^. These initial studies indicate that stabilized mRNAs can produce equivalent, and for some designs, more protein compared with non-optimized mRNAs^13^. These design strategies are predicted to be able to produce mRNAs that do not activate double-stranded RNA immune sensors^12^ such as RIG-I^14^. These strategies have also demonstrated compatibility with mRNAs synthesized from modified nucleotides including pseudouridine^13^, which are used in mRNA vaccine formulations^15^.

However, the potential of any such mRNA design algorithm is limited by the accuracy of the underlying model in predicting RNA degradation. Previous models for RNA degradation have assumed that the probability of any RNA nucleotide linkage being cleaved is proportional to the probability of the 5′ nucleotide being unpaired^12^. Computational studies with this model suggested that at least a twofold increase in stability could be achieved through sequence design, while maintaining a wide diversity of sequences and features related to translatability, immunogenicity and global structure^13^. However, it is unlikely that degradation depends only on the probability of a nucleotide being unpaired: local sequence- and structure-specific contexts may vary widely, as evidenced by ribozyme RNAs found in nature, whose sequences adopt specific structures that undergo self-scission^16^.

**Fig. 1:**
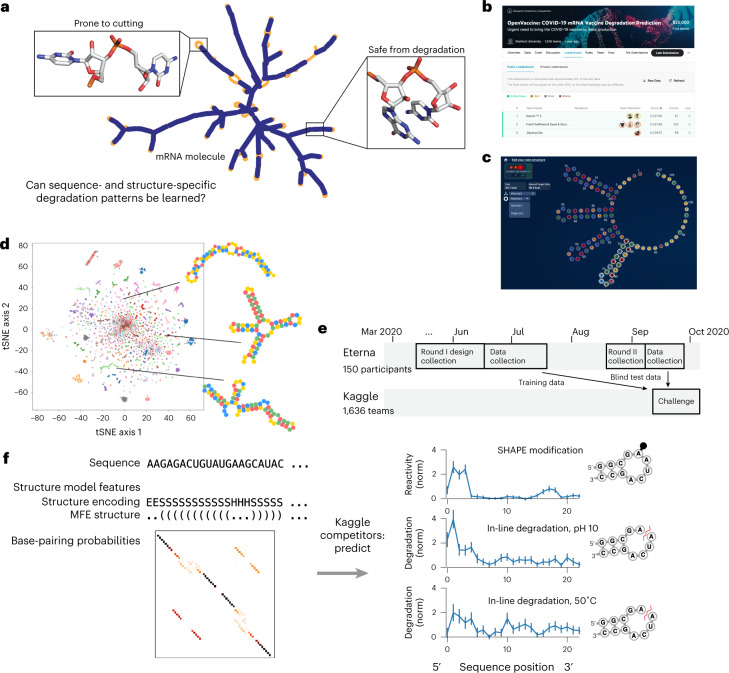
Dual-crowdsourcing setup for creating predictive models of RNA degradation. **a**, mRNA molecules fold into secondary structures containing unpaired regions prone to hydrolysis and limiting to therapeutic stability. **b**, Screenshot of the OpenVaccine Kaggle competition public leaderboard. **c**, Screenshot of an example construct designed by an Eterna participant in the ‘Roll Your Own Structure’ challenge (‘rainbow tetraloops 7’ by Omei). **d**, tSNE^38^ projection of training sequences of ‘Roll Your Own Structure’ Round I, marker style and colours indicating 150 Eterna participants. Lines indicate example short 68 nt RNA fragments. **e**, Timelines of dual-crowdsourced challenges. Eterna participants designed datasets that were used for training and blind test data for Kaggle machine learning competition to predict RNA chemical mapping signal and degradation. **f**, Kaggle participants were given RNA sequence and structure information and asked to predict RNA degradation profiles and SHAPE reactivity. In-structure encoding features, S = stem, H = hairpin, E = end, etc. from bpRNA^24^. Data are presented as mean ± standard deviation estimated from Poisson counting error in sequencing reads (*n* = 1 biologically independent sample).

We wished to understand the maximum predictive power achievable for RNA degradation on a short timescale for model development. To do this, we combined two crowdsourcing platforms: Eterna, an RNA design platform, and Kaggle, a platform for machine learning competitions. The problem of ‘RNA design’ involves designing RNA sequences with specific target properties such as a particular overall structure^17,18^, a target function such as sensor activity^19^, or, in this case, high chemical stability^13^. We used degradation data from short RNA fragments designed on the Eterna platform, which comprised a wide diversity of sequences and structures, and hypothesized that crowdsourcing the problem of obtaining a machine learning architecture would result in a model capable of expressing the resulting complexity of sequence- and structure-dependent degradation patterns (Fig. 1a). We hypothesized that this ‘dual crowdsourcing’ would lead to stringent and independent tests of the models developed, minimizing sharing of assumptions between the individuals designing the constructs to test (Eterna participants) and the individuals building the models (Kaggle participants) and leading to better generalizability on independent datasets.

The resulting models were subjected to two blind prediction challenges. The first was in the context of the Kaggle competition, where the RNA structure probing and degradation data that participants would be aiming to predict was not acquired until after the competition was announced. The experimental method used for these data, In-line-seq, allowed for measuring the degradation rate of individual nucleotide linkages. However, this method relies on probing short RNA fragments and is unable to scale to make single-nucleotide degradation measurements of full-length mRNAs for protein targets of interest. Other experimental methods such as PERSIST-seq^13^ have been developed to characterize the overall degradation rates per mRNA molecule, which is the primary value of interest to minimize when designing stabilized RNA-based therapeutics. In principle, the overall degradation rate of an mRNA molecule of length *N* is equivalent to the sum of degradation rates at each dinucleotide linkage in the backbone^12^:

1 $$k_\mathrm{deg}^\mathrm{mRNA} = \mathop {\sum}\nolimits_{i = 1}^{N - 1} {k_\mathrm{deg}^i,}$$

where $k_\mathrm{deg}^i$ is the degradation of nucleotide linkage *i*. The half-life of the mRNA is calculated as

2 $$t_{1/2} = \frac{{\ln 2}}{{k_\mathrm{deg}^\mathrm{mRNA}}}.$$

**Extended Data Fig. 1:**
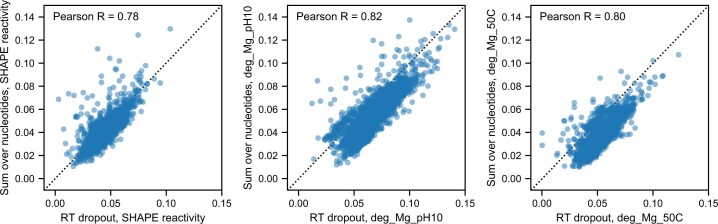
Summed degradation rates and overall degradation rate are highly correlated. Summed per-nucleotide degradation rates and overall degradation rate, estimated by log(abundance of full-length construct), are highly correlated in Rounds 1 and 2. Source data

We tested the above model empirically by comparing the summed degradation rates per nucleotide to the abundance of the entire construct remaining from sequencing and found high agreement (Extended Data Fig. 1). Using the above ansatz, the resulting models were tested in a second blind challenge of predicting the overall degradation of full-length mRNAs encoding a variety of model proteins, experimentally tested using PERSIST-seq. The models also demonstrated increased predictive power over existing methods in predicting these overall degradation rates. These models therefore appear immediately useful for guiding design of low-degradation mRNA molecules. Analysis of model performance suggests that the task of predicting RNA degradation patterns is limited by both the amount of data available as well as the accuracy of the structure prediction tools used to create input features. Further developments in experimental data and secondary structure prediction, when combined with network architectures such as those developed here, will further advance RNA degradation prediction and therapeutic design.

## Results

### Dual-crowdsourced competition design and assessment

The aim of the OpenVaccine Kaggle competition (Fig. 1b) was to develop computational models for predicting RNA degradation patterns. We asked participants on the Eterna platform to submit RNA designs using a web-browser design window (Fig. 1c), which resulted in a diversity of sequences and structures (Fig. 1d). In total, 150 participants (Supplementary Table 1) submitted sequences. A secondary motivation was an opportunity for participants to receive feedback on RNA fragments they may wish to use in mRNA design challenges described by Leppek et al.^13^ In total, 3,029 RNA designs of length 107 nt were collected in the first ‘Roll Your Own Structure’ round I (RYOS-I), which opened on 26 March 2020 and closed on 19 June 2020 (Fig. 1e).

We then obtained nucleotide-level degradation profiles for the first 68 nucleotides of these RNAs using In-line-seq^13^, a method for characterizing in-line RNA degradation in high throughput for the purposes of designing stabilized RNA therapeutics. In brief, a library of short RNA fragments was produced from a DNA library via in vitro transcription, each of which contained a unique barcode at the 3′ terminus. The RNA library was subjected to one of several accelerated degradation conditions, which included combinations of increases in Mg^2+^ concentration, basicity and temperature. The resulting fragmented RNA was reverse transcribed and the complementary DNA was sequenced. The base-pairing structures of the constructs were also characterized via selective 2′ hydroxyl acylation with primer extension (SHAPE; termed ‘Reactivity’ below)^20,21^, a technique to characterize RNA secondary structure. SHAPE experiments were performed analogously to the In-line-seq experiments described above, but instead of degradation conditions, the RNA was subjected to a chemical modifier (1-methyl-7-nitroisatoic anhydride, 1M7) which acylates the 2′-OH group. When the RNA is reverse transcribed, such an acylation causes the reverse transcriptase enzyme to terminate. The resulting cDNA fragments were used to create a ‘reactivity profile’ for each molecule.

The Kaggle competition was designed to create models that would have predictive power for three of these data types, given RNA sequence and secondary structure as input (Fig. 1f). In addition to scoring two types of degradation data, we also scored predictions for SHAPE data, hypothesizing that models would be more accurate if able to learn shared underlying features between degradation data and SHAPE data as a form of multi-task training. Nucleotides that are more reactive to the SHAPE reagent would be predicted to also have dinucleotide linkages with higher degradation rates.

In total, each independent construct of length *N* required predicting 3 × *N* values for the three data types. In addition to these experimental data, Kaggle participants were also provided with features related to RNA secondary structure computed from available biophysical models to use if they wished. These features included N × N base-pairing probability matrices from EternaFold^22^, a recently developed package with state-of-the-art performance on RNA structural ensembles; dot-parenthesis notated minimum free energy (MFE) RNA secondary structure from the ViennaRNA package^23^; and a six-character featurization of the MFE structure calculated using bpRNA^24^.

**Fig. 2:**
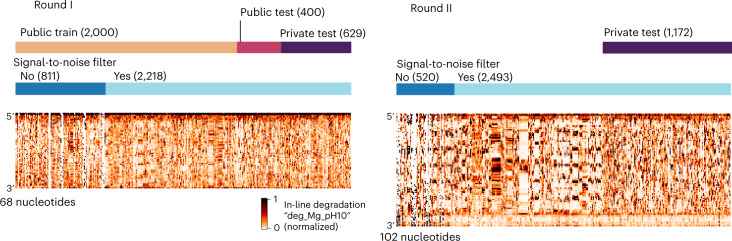
Signal-to-noise filtering and hierarchical clustering was used to filter the constructs designed by Eterna participants to create a test set of constructs that were maximally distant from other test constructs. Heatmaps of data type ‘Deg_Mg_pH10’ (10 mM Mg^2+^, pH 10, 1 day, 24˚C).

We developed training and ‘public test’ datasets from the RYOS-I dataset (Fig. 2). The public test dataset was used to rank submissions during the competition. The 3,029 constructs were filtered for those with mean signal-to-noise values greater than 1, resulting in 2,218 constructs (Fig. 2, dark blue track, Methods). These constructs were segmented into splits of 1,179 in the public training dataset, 400 constructs in the public test set and 639 for the ‘private test’ dataset, the set which would be used in the final evaluation. The sequences that did not pass the signal-to-noise filter were also provided to Kaggle participants with the according description. The RYOS-I data contained some ‘clusters’ of sequences where Eterna players included many small variations on a single sequence (clusters visible in Fig. 1d). To mitigate the possibility of sequence motifs in these clusters biasing evaluation, we segmented the RYOS-I data into a public training, public test and private test sets by clustering the sequences and including only sequences that were singly, doubly or triply clustered in the private test set (Methods). This strategy was described to Kaggle participants during the competition.

To ensure that the majority of the data used for the private test set were fully blind, we initiated a second ‘Roll Your Own Structure’ challenge that was launched for Eterna design collection on 18 August 2020. Given that useful models for degradation should be agnostic to RNA length, we designed the constructs in RYOS-II to be 34 nucleotides longer (102 versus 68 nt) than the constructs in RYOS-I to discourage modelling methods that would overfit to constructs of length 68. Design collection was closed on 7 September, 3 days before the launch of the Kaggle challenge on 10 September. The RYOS-II wet-lab experiments were conducted concurrently with the Kaggle challenge, enabling a completely blind test for the models developed on Kaggle. The Kaggle competition was closed on 6 October. The RYOS-II was similarly clustered and filtered to ensure that the test set used for scoring consisted primarily of singly and doubly clustered constructs. Three data types were used to score models: SHAPE; 10 mM Mg^2+^, pH 10, 1 day, 24 °C; and 10 mM Mg^2+^, pH 7.2, 1 day, 50 °C. Models were scored using the mean column RMSE (MCRMSE) across three data types, defined as

3 $$\mathrm{MCRMSE} = \frac{1}{{N_t}}\mathop {\sum}\nolimits_{j = 1}^{N_t} {\sqrt {\frac{1}{n}\mathop {\sum}\nolimits_{i = 1}^n {\left( {y_{ij} - \hat y_{ij}} \right)^2} } ,}$$

where *N*_*t*_ is the number of scored data types, *n* is the number of nucleotides in the dataset, *y*_*ij*_ is the measured data value, and $\hat y_{ij}$ is the predicted data value for nucleotide *i* in sequence *j*. Two additional data types were included in the training data corresponding to RNAs degraded for 7 days without Mg^2+^ rather than 1 day with Mg^2+^: (pH 10, 7 days, 24 °C; and pH 7.2, 7 days, 50 °C). However, these data were not collected for the second round to accelerate competition turnaround.

### Kaggle team performance and common attributes of top models

**Fig. 3:**
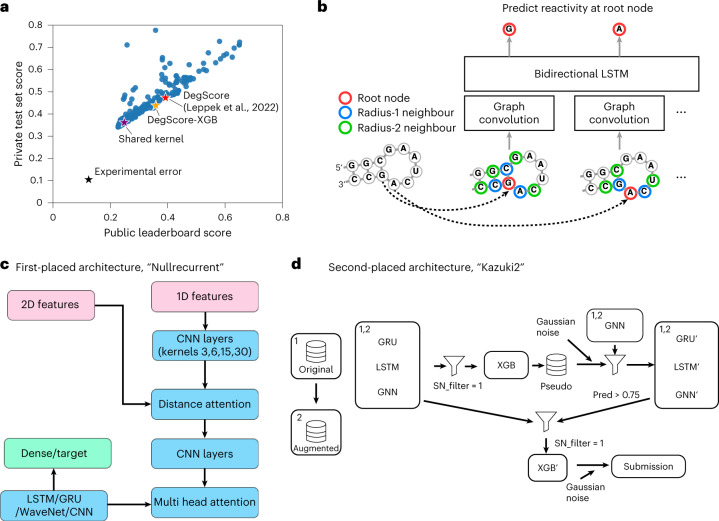
Deep learning strategies used in competition. **a**, Public test versus private test performance of all teams in the Kaggle challenge. Black star: experimental error. Red star: DegScore baseline model^13^. Orange star: DegScore-XGB model using DegScore featurization with XGBoost. Purple star: baseline kernel used by many top-performing teams. **b**, Distance embedding used to represent nucleotide proximity to other nucleotides in secondary structure. **c**, Schematic of the single neural net (NN) architecture used by the first-placed solution. This solution combined two sets of features into a single NN architecture, which combined elements of classic recurrent neural networks and convolutional neural networks. **d**, Schematic of the full solution pipeline for the second-placed solution. This solution combined single-model neural networks, similar to the ones used for the first-placed solution, with more complex second- and third-level stacking using XGBoost^25^ as the higher level learner. Abbreviations in schematics: CNN: convolutional neural network, GRU: gated recurrent unit, GNN: graph neural network, LSTM: long short-term memory neural network, SN: signal-noise, XGB: XGBoost. Source data

**Extended Data Fig. 2:**
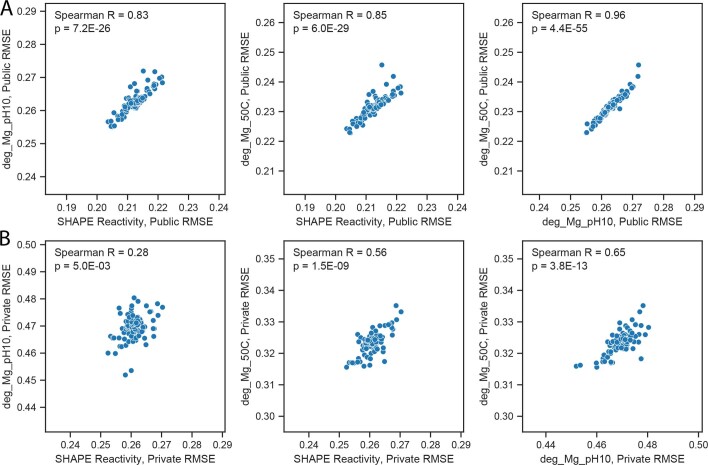
Correlations between data types across models. Correlation between RMSE by data type for the top 100 teams for the **(A)** public and **(B)** private test sets. Significance test for Spearman correlation value is two-sided p-value for a hypothesis test whose null hypothesis is that two sets of data are uncorrelated. Source data

**Extended Data Fig. 3:**
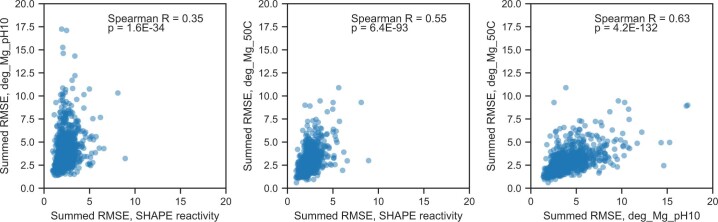
Correlations between data types across constructs for top model. Correlation between RMSE by data type across constructs from the 1^st^ place ‘Nullrecurrent’ model on the private test set. Significance test for Spearman correlation value is two-sided p-value for a hypothesis test whose null hypothesis is that two sets of data are uncorrelated. Source data

During the 3 week competition period, 1,636 teams submitted 35,806 solutions. The overall performance of teams compared to baseline models for RNA degradation is depicted in Fig. 3a. Kaggle entries significantly outperformed the ‘DegScore’ linear regression model for RNA degradation^13^ by 37% in MCRMSE for the public test set and 25% for the private test set (Fig. 3a). We found that for predictions from the top 100 teams (Extended Data Fig. 2) as well as amongst predictions between individual constructs (Extended Data Fig. 3), performance between data types was highly correlated in the public dataset and less strongly correlated in the private dataset. Overall, the weakest correlation was between SHAPE and degradation data types.

An additional benchmark model that used the DegScore windowed featurization (see Methods) with improved XGBoost^25^ training, termed the DegScore-XGB model, resulted in moderate improvement (public MCRMSE 0.35854, private MCRMSE 0.43850 compared to public MCRMSE 0.39219, private MCRMSE 0.47197 for the original DegScore). Kaggle participants developed feature encodings beyond what was provided. One of the most widely used community-developed featurizations was a graph-based distance embedding depicted in Fig. 3b. Several teams, including the top three teams, used a publicly shared autoencoder/GNN/GRU kernel (https://www.kaggle.com/code/mrkmakr/covid-ae-pretrain-gnn-attn-cnn/), which alone achieved a MCRMSE of 0.24860 on the public and 0.36106 on the private test set (Fig. 3a). This notebook was the most forked (forked 936 times as of March 2022) and upvoted (upvoted 386 times). The architecture of the winning ‘Nullrecurrent’ model (Fig. 3c) depicts the architecture of this shared kernel, which feeds 1D and 2D features, including adjacency matrices based on the secondary structure of the RNA inputs, into a multi-head attention network, the output of which is then fed into convolutional neural net layers. Many teams additionally cited pseudo-labelling and generating additional mock data as being integral to their solutions. The architecture of the second-placed team (Fig. 3d) demonstrates an example implementation of using pseudo-labelling. The practice of pseudo-labelling, which is similar to the student-teacher learning paradigm,^26^ involves using predictions from one model as ‘mock ground truth’ labels for another model. To generate additional mock data, participants generated random RNAs and structure featurizations from five different secondary structure prediction algorithms using the package Arnie (https://github.com/DasLab/arnie) and used these RNAs in training as well (see Supplementary Information for more detailed descriptions of solutions from Kaggle teams).

We explored whether increased accuracy in modelling could be achieved by ensembling models, that is, combining predictions from multiple models; a common feature of Kaggle competitions is that winning solutions are dissimilar enough that ensembled models frequently improve predictive ability. We found that ensembling resulted in only modest improvements (Methods), suggesting the majority of signal had been captured by the top two models.

### Top models are capable of deep representation of RNA motifs

We analysed predictions from the first-placed model (‘Nullrecurrent’) in greater depth to better understand its performance. Across all nucleotides in the private test set, 41% of nucleotide-level predictions for SHAPE reactivity agreed with experimental measurements with an error margin that was lower than experimental uncertainty; for comparison, if experimental errors are distributed as normal distributions, a perfect predictor would agree with experimental values over 68% of data points. For Deg_Mg_pH10 and Deg_Mg_50C, 28% and 42% of predictions were within error, respectively.

**Fig. 4:**
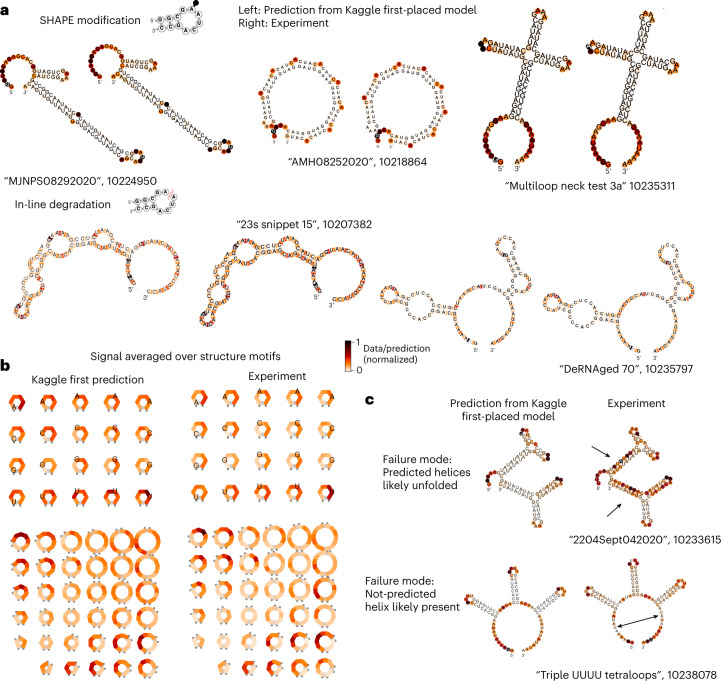
Deep-learning models can represent RNA-structure-based observables. **a**, Representative structures from the best-predicted constructs from SHAPE modification (top row) and degradation at 10 mM Mg^2+^, pH 10, 1 day, 24 °C (Deg_Mg_pH10, bottom row). **b**, Nullrecurrent model predictions and experimental signal, averaged over secondary structure motifs. **c**, One failure mode for prediction came from constructs whose input secondary structure features were possibly incorrectly predicted.

**Extended Data Fig. 4:**
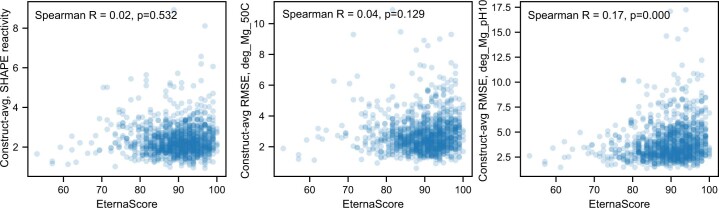
Comparing model error and EternaScore. RMSE of Nullrecurrent model did not significantly correlate with EternaScore, a measure of how closely the SHAPE reactivity data matched the predicted secondary structure. Significance test for Spearman correlation value is two-sided p-value for a hypothesis test whose null hypothesis is that two sets of data are uncorrelated. Source data

The nucleotides with the highest RMSE in the Deg_Mg_pH10 data type were any nucleotide type in bulges, and U’s in any unpaired context. Figure 4a depicts representative constructs with the lowest RMSE for the Deg_Mg_pH10 data type out of the private test data, demonstrating that diverse structures and structure motifs were capable of being predicted correctly. Aggregating predictions from the Nullrecurrent model over secondary structure motifs (Fig. 4b) demonstrates that the Nullrecurrent model captured patterns previously observed in the experimental signal^13^. The most reactive RNA structure motifs were triloops, a previously unknown biological finding. Another unexpected finding from these data was that symmetric internal loops were more stable against degradation than internal loops with asymmetric lengths. The fact that the Nullrecurrent model was able to capture this trend indicates that using such models within a design algorithm would allow for an automated way to model such biochemical attributes within a designed mRNA. Constructs with the highest RMSE highlight instances in which the provided structure features were incorrect. Figure 4c depicts two constructs with the highest RMSE for the SHAPE modification prediction. The SHAPE data for the first construct, ‘2204Sept042020’, has high reactivity in predicted stem areas, indicating that the stems were unfolded in solution. By contrast, construct ‘Triple UUUU Tetraloops’ has low reactivity in the exterior loop, suggesting that those nucleotides were paired rather than unpaired. These examples notwithstanding, we found no correlation between the EternaScore, a metric indicating how closely the experimental reactivity signal matches the predicted structure^17^, and RMSE summed per construct, suggesting that, in general, quality of the input structure features was not a limitation in model training (Extended Data Fig. 4).

### Kaggle models improve prediction of mRNA degradation

**Fig. 5:**
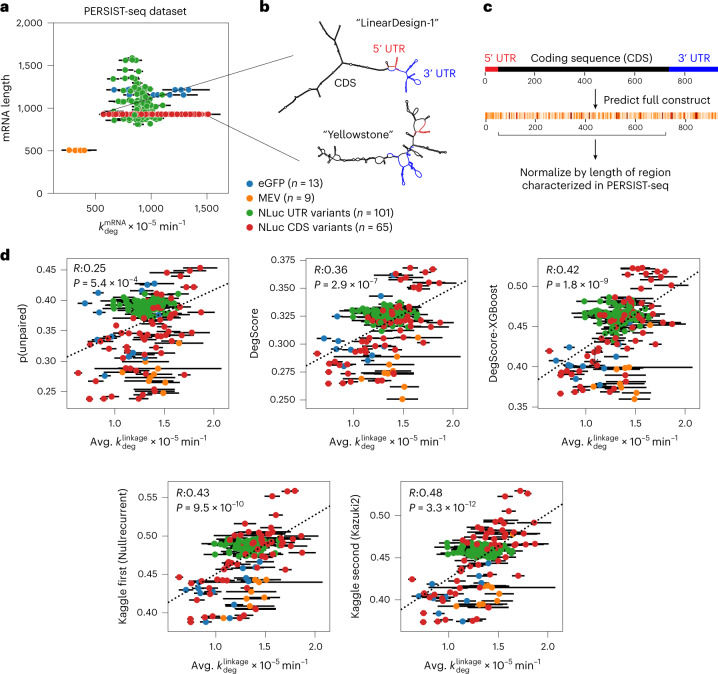
Kaggle models demonstrate improved performance in independent test of degradation of full-length mRNAs. **a**, Overall mRNA degradation rate from PERSIST seq is driven by mRNA length. Kaggle models were therefore tested in their ability to predict length-averaged mRNA degradation. Data are presented as mean values ± standard error estimated from the PERSIST-seq experiment, *n* = 3 biologically independent samples. **b**, Representative structures of two mRNAs of the same length that both encode nanoluciferase, one with high degradation (‘Yellowstone’, left) and low degradation (‘LinearDesign-1’, right). These mRNAs were designed by Eterna participants, and were used as a negative control and positive control of structured mRNA in ref. ^13^. **c**, Prediction vectors were summed over nucleotides corresponding to the CDS region to compare to PERSIST-seq degradation rates, which account for degradation between two RT-PCR primers designed to capture degradation in the CDS region. **d**, Length-normalized predictions from the Kaggle first-placed ‘Nullrecurrent’ model and Kaggle second-placed ‘Kazuki2’ model show improved prediction over unpaired probabilities from ViennaRNA RNAfold^23^ and the DegScore linear regression model^13^, and a version of the DegScore featurization with XGBoost^25^ training. Data are presented as mean values ± standard error estimated from the PERSIST-seq experiment, *n* = 3 biologically independent samples. Significance test for Spearman correlation value is two-sided *p*-value for a hypothesis test whose null hypothesis is that two sets of data are uncorrelated. Source data

As an independent test, we assessed the ability of the top two Kaggle models to predict the overall degradation rates for a dataset of full-length mRNAs that were not publicly available at the time of the Kaggle competition. Because the throughput of the In-line-seq experimental method is limited to RNA lengths easily accessible by Illumina sequencers (500 nt), these mRNAs could not be probed at a per-nucleotide level akin to the datasets used in the Kaggle experiments. However, their overall degradation rates (related to the per-nucleotide degradation rate via equation (1)), were characterized using PERSIST-seq^13^. In brief, the PERSIST-seq technique measured the overall degradation rate of a mRNA by monitoring the mRNA’s abundance using reverse transcription followed by polymerase chain reaction amplification (RT-PCR) at varying timepoints after degradation was initiated. The lengths of these mRNAs ranged from 504 to 1,588 with a median length of 928 (Fig. 5a), nearly tenfold longer than the longest RNA fragments used in the OpenVaccine Kaggle challenge (full mRNA dataset, attributes and calculations in Supplementary Table 2). The experimentally determined structures of two example mRNAs designed by Eterna participants^13^ are depicted in Fig. 5b. Both code for Nanoluciferase but have a 2.5-fold difference in hydrolysis lifetime. ‘Yellowstone’ was designed by an Eterna participant using codons that mimic nucleotide frequencies from organisms in Yellowstone hot springs^27^; ‘LinearDesign-1’ was designed by an Eterna participant using an initial sequence from the LinearDesign mRNA structure optimization server^28^.

To compare the Kaggle predictors to the single overall degradation rate from PERSIST-seq, we made predictions for all nucleotides in the full mRNA constructs and summed the predictions from the region that was captured by RT-PCR in PERSIST-seq which, in most cases, included the mRNA’s 5′ untranslated region (UTR) and coding sequence (CDS) (Fig. 5c). Carrying out predictions on the full RNA sequence and then summing over the probed window accounts for interactions between the untranslated regions and CDS, as can be seen for two example constructs in Fig. 5b–nucleotides in the 5′ and 3′ UTRs are predicted to pair with the CDS. We made predictions for 188 mRNAs in four classes of protein targets: a short multi-epitope vaccine (MEV), the model protein nanoluciferase, with one class consisting of varied UTRs and a second consisting of varied CDSs, and enhanced green fluorescent protein (eGFP). We found that the Kaggle second-placed ‘Kazuki2’ model exhibited the highest correlation to experimentally determined degradation rates, followed by the Kaggle first-placed ‘Nullrecurrent’ model (Fig. 5c), with Spearman correlation coefficients of 0.48 (*p* = 3.3 × 10^–12^) and 0.43 (*p* = 9.5 × 10^–^^10^), respectively. Both Kaggle models outperformed unpaired probability values from ViennaRNA RNAfold v. 2.4.14^23^ (*R* = 0.25, *p* = 5.4 × 10^–^^4^), the DegScore linear regression model (*R* = 0.36, *p* = 2.9 × 10^–^^7^) and the DegScore-XGBoost model (*R* = 0.42, *p* = 1.8 × 10^–^^9^). An ensemble of the Nullrecurrent and Kazuki2 models did not outperform the Kazuki2 model (*R* = 0.47, *p* = 1.4 × 10^–^^11^), again suggesting that the models themselves had reached their predictive potential. To estimate an upper limit for correlation considering experimental error, we resampled the measured degradation rates from within experimental error and calculated the correlation to the mean degradation rate. This resulted in a Spearman correlation of 0.88 (Table 1).

**Table 1:** Results from models tested in this work on Kaggle OpenVaccine public leaderboard, private test set and orthogonal mRNA degradation results

	Public test set (400 constructs, 27,200 nt)	Private test set (1,801 constructs, 162,316 nt)	mRNA degradation prediction from ref. ^6^ (188 constructs)
**Metric**	MCRMSE	MCRMSE	Spearman correlation
Experimental error	0.12491	0.10571	0.88^a^
**Single model (blind prediction)**			
DegScore	0.39219	0.47297	0.36
DegScore-XGBoost	0.35854	0.43850	0.42
Nullrecurrent	0.22758	**0.34198**	0.43
Kazuki2	**0.22756**	0.34266	**0.48**
**Ensembled models (post hoc)**			
Genetic algorithm (10 of top 100 selected)	**0.2237**	0.3397	
Ensemble top two models	0.2244	**0.33788**	0.47
Genetic algorithm on private test set		0.3382	–

^a^Spearman correlation of experimental length-normalized degradation rate,resampled from experimental error.

## Discussion

The OpenVaccine competition uniquely leveraged resources from two complementary crowdsourcing platforms: Kaggle and Eterna. The participants in the Kaggle competition were tasked with predicting stability measurements of individual RNA nucleotides. The urgency of timely development of a stable COVID-19 mRNA vaccine necessitated that the competition be run on a relatively short timeframe of three weeks, as opposed to three months, which is more common with Kaggle competitions.

The models presented here are immediately useful for mRNA design in that they could be called within a stochastic mRNA design algorithm^12^ to minimize the predicted degradation. There is likely further opportunity to leverage advancements in natural language processing to use datasets such as the ones presented here to generate mRNA designs using text-generation approaches^29–31^. The degradation data used in this competition were from RNA synthesized with unmodified nucleotides, but mRNA vaccines are being formulated with modified nucleotides including pseudouridine or *N*-1-methyl-pseudouridine^15^. Modified nucleotides in general will have differing underlying thermodynamics^32^, and there is a need to develop datasets and predictive models to predict structures and resulting stabilization of mRNAs formulated with modified nucleotides. The In-line-seq method can be performed using RNA with modified nucleotides, and the resulting data could be used to re-train models with architectures such as the ones presented here. Short of developing complete new thermodynamic parameters for modified nucleotides, it may be possible to develop principled heuristics to adapt models to mRNAs synthesized with modified nucleotides. For instance, Leppek et al. modified the DegScore model for pseudouridine by setting all uridine degradation measurements to zero to mimic the stabilization effect of pseudouridine, and saw moderate improvement in correlation^13^.

Kaggle competitions with relatively small datasets can be subject to serious overfitting to the public leaderboard, which often leads to a ‘shake-up’ of the leaderboard when the results on the unseen test set are announced. In this competition the shake-up was minimal—most of the top teams were ranked close to the same position on the private leaderboard as they were on the public leaderboard. As the private leaderboard was determined on data that had not been collected at the time of the competition launch, this result suggests that the models are robust and generalizable. We demonstrated the top two models generalized to the task of predicting degradation for full-length mRNA molecules that were tenfold longer than the constructs used for training. We speculate that the use of a separate, independently collected dataset for the private leaderboard tests—a true blind prediction challenge—was important for ensuring generalizability. The winning solutions all combined neural network architectures that are commonly used in modelling 1D sequential data, including multihead attention, recurrent NNs (LSTMs and GRUs) and 1D CNNs. The effectiveness of pseudo-labelling has two implications: more data will likely benefit any future modelling efforts, and the simple architectures that were used have enough capacity to benefit from more data.

**Extended Data Fig. 5:**
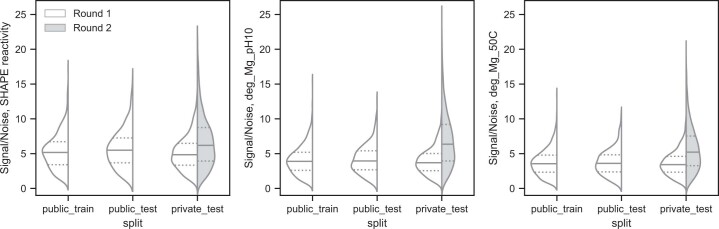
Signal-noise of data splits. Average signal-noise from data splits, separated by data from Rounds 1 and 2. Solid lines: median, dotted lines: 25/75% percentile.

An under-investigated aspect of the models presented here is the effect of training on multiple data types. We speculate that because SHAPE reactivity has higher signal-to-noise than the degradation data types (Extended Data Fig. 5), models with architectures that allowed for weight-sharing between data types benefitted from learning to predict SHAPE reactivity as well. Directly predicting RNA degradation without concurrently training on SHAPE data may result in worse model performance. Conversely, the model architectures presented here may also prove to have useful biological applications in predicting only SHAPE reactivity data. Future directions for model development include training such models on larger chemical mapping datasets from more diverse experimental sources^22^ and integrating into inference frameworks for RNA structure prediction^22,33^.

Finally, the models for predicting RNA hydrolysis developed in this work may prove useful in computationally identifying classes of natural RNAs that have evolved to be resistant to degradation^34^. Such future bioinformatic analysis may suggest entirely new biologically inspired approaches for designing hydrolysis-resistant RNA therapeutics. More immediately, it will be of strong interest to computationally design mRNA sequences that optimize the predicted degradation stability discovered in this study, and to experimentally test if such sequences are indeed sufficiently stable to enable wider distribution of mRNA vaccines. In silico design of neural-network-predicted properties is an active area of research, and we speculate that further dual-crowdsourcing studies may help accelerate progress.

## Methods

### Initial feature generation

As a starting point for Kaggle teams, we supplied a collection of features for each RNA sequence, including the minimum free energy (MFE) structure according to the ViennaRNA 2 energy model^23^, ‘loop type’, or secondary structure type assignments generated with bpRNA^24^ (S = Stem, I = Internal loop, B = Bulge, H = Hairpin, M = Multiloop, X = external loop, E = end, terminology adopted from bpRNA) and the base-pair probability matrix according to the EternaFold^22^ energy model. These features were generated using Arnie (https://github.com/DasLab/arnie).

### Experimental data generation

The first experimental dataset used in this work, for the public training and test set, resulted from the ‘Roll-Your-Own-Structure’ Round I lab on Eterna, and had been generated previously by Leppek et al.^13^

The second experimental dataset used in this work, for the private test set, was generated for this work specifically. To produce these data, and for precise consistency with the public training and test set, In-line-seq was carried out as described by Leppek et al.^13^ In brief, DNA templates were ordered via custom oligonucleotide pool from Custom Array/Genscript, prepended by the T7 RNA polymerase promoter. Templates were amplified via PCR, transcribed to RNA via the TranscriptAid T7 High Yield Transcription Kit (Thermofisher, K0441), and the purified RNA was subjected to degradation conditions: (1) 50 mM Na-CHES buffer (pH 10.0) at room temperature without added MgCl_2_; (2) 50 mM Na-CHES buffer (pH 10.0) at room temperature with 10 mM MgCl_2_; (3) phosphate-buffered saline (PBS, pH 7.2; Thermo Fisher Scientific-Gibco 20012027) at 50 °C without added MgCl_2_; and (4) PBS (pH 7.2) at 50 °C with 10 mM MgCl_2_. In parallel, purified RNA was subjected to SHAPE structure-probing conditions, and one sample was subjected to the SHAPE protocol absent addition of the 1-methyl-7-nitroisatoic anhydride reagent.

cDNA was prepared from the six RNA samples (SHAPE probed, control reaction and four degradation conditions). We pooled 1.5 μl of each cDNA sample together, ligated with an Illumina adapter, washed and resuspended the ligated product, which was quantified by qPCR, sequenced using an Illumina Miseq. Resulting reads were analysed using MAPseeker (https://eternagame.org/software) following the recommended steps for sequence assignment, background subtraction of the no-modification control, correction for signal attenuation and reactivity profile normalization as described previously^20^.

### Signal-to-noise filtering

**Extended Data Fig. 6:**
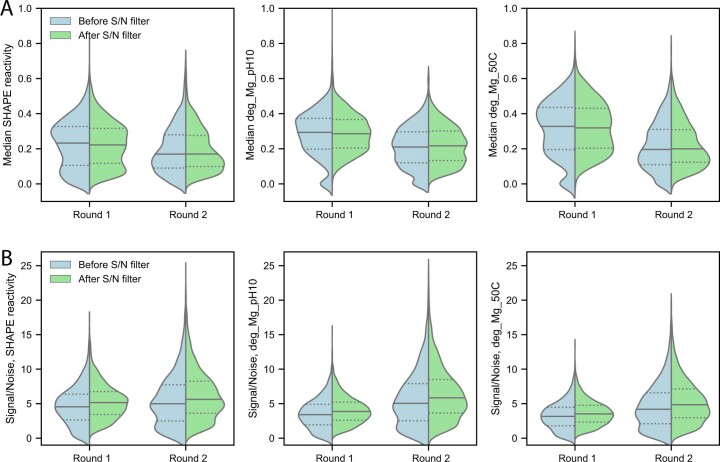
Effect of signal-noise filter on data distributions. Effect of signal-noise filter (described in Methods) on **(A)** median reactivity/degradation per construct and **(B)** average signal-noise per construct for Rounds 1 and 2. Solid lines: median, dotted lines: 25/75% percentile.

Data were filtered to include RNAs with a minimum value >0.5, maximum value <20 across five RNA degradation conditions and RNAs with a signal/noise ratio for SHAPE reactivity greater than 1.0. Signal/noise ratio for each construct was calculated as

4 $${\mathrm{SN}}\;{\mathrm{ratio}} = \frac{1}{M}\mathop {\sum}\nolimits_{i = 1}^M {\frac{1}{N}\mathop {\sum}\nolimits_{j = 1}^N {\frac{{\mu _{i,j}}}{{\sigma _{i,j}}}} } ,$$

where *μ*_*i*,*j*_ is the mean value of data type *i* at nucleotide *j*, and *σ*_*i*,*j*_ is standard deviation of data type *i* at nucleotide *j*, as calculated by MAPseeker. The data that did not pass the above filters were also provided to participants to give the option to use in training, and was flagged with the variable ‘SN_filter = 0’. Applying the above filter did not significantly alter the distribution of the median reactivity or signal/noise of any data type (SHAPE reactivity, Deg_Mg_pH10, Deg_Mg_50C) within either dataset (RYOS 1 or RYOS 2; Extended Data Fig. 6). However, average signal/noise of the Round II constructs was higher than the Round 1 constructs. Average signal/noise ratio for SHAPE reactivity across each dataset increased from 5.3±2.4 (mean ± standard deviation) to 6.2±3.5; for deg_Mg_pH10, 4.1±2.0 to 6.4±3.8 for Rounds 1 and 2; and for deg_Mg_50C 3.87±1.8 to 5.3±3.1 (Extended Data Fig. 5).

**Extended Data Fig. 7:**
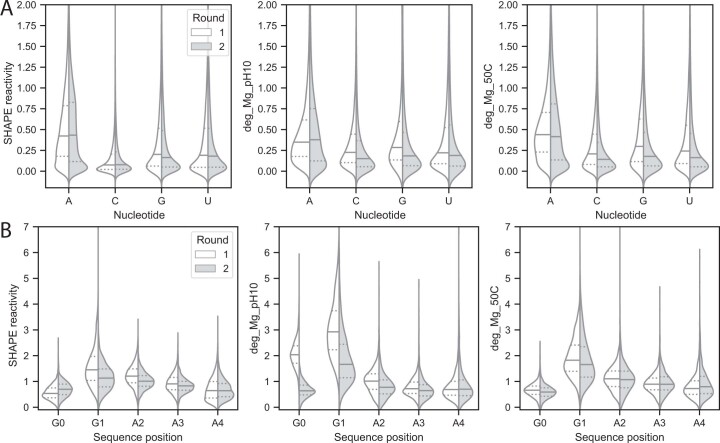
Data disaggregated by nucleotide. **(A)** Reactivity/degradation data disaggregated by nucleotide from rounds 1 and 2, from constructs that passed signal-noise filter. **(B)** Reactivity/degradation data from first 5 nucleotides from rounds 1 and 2, which were a constant ‘GGAAA’. Solid lines: median, dotted lines: 25/75% percentile.

We wished to ascertain if the measured reactivities and degradation from Round 2 needed to be rescaled to match Round 1. To assess this, we compared distributions of nucleotide reactivities from nucleotide types. We found that for each data type and nucleotide type, the median values for Round 2 were within the 50% interquartile range of Round 1 (Extended Data Fig. 7a). We also compared distributions of reactivity from the first five nucleotides, which are a constant ‘GGAAA’ for each construct. The median values for each nucleotide in this were within the 50% interquartile range for all except for the first two GG’s in the ‘Deg_Mg_pH10’ data type (Extended Data Fig. 7b). We elected to not rescale the data from Round 2.

### Private test set curation

The private test set was curated to avoid bias toward more highly represented sequence motifs from the Eterna designs. Sequences that passed the above filters were clustered hierarchically using the ‘ward’ method in scikit-learn^35^ and then clustered at a cophenetic distance of 0.5. That is, sequences within the same cluster have <50% sequence similarity. All sequences that were in clusters with one, two or three members were included in the private test set, as well as one cluster member from other randomly selected clusters to attain the desired number of test set constructs.

### Comparing to the DegScore model

We compared Kaggle models to the ‘DegScore’ linear model^13^, which models degradation at a given nucleotide *i* as a linear function of nucleotides surrounding *i*:

5 $$\begin{array}{l}Y_i = \mathop {\sum}\nolimits_{k = - w}^w \left[ \mathop {\sum}\nolimits_{n \in \{\mathrm{A,C,G,U}\} } \left( \beta _{k,n}I_{i + k,n} \right) \right] \\+ \mathop {\sum}\nolimits_{k = - w}^w \left[ \mathop {\sum}\nolimits_{s \in \{\mathrm{H,E,I,M,B,S}\} } \left( {\beta _{k,s}I_{i + k,s}} \right) \right] + \beta _0 ,\end{array}$$

where *β* represents learned coefficients and *I* is an indicator function corresponding to the identity of nucleotide *i+k. I* accounts for sequence identity *n*(A,C,G,U) and its secondary structure type assignment *s* (S = stem, E = external loop, I = internal loop, B = bulge, H = hairpin, M = multiloop). The secondary structure type assignment ‘X’ for external loop was replaced with ‘E’ in the DegScore model in ref. ^13^, to reflect the biophysical similarity between the two categories. *w* is the maximum window distance, set to be 12 by Leppek et al.^13^. For a window size of *w* = 12, there are 251 parameters (25 positions with 4 sequence indicators and 6 secondary structure indicators for each position, and 1 intercept parameter).

### Ensembling models

We explored whether increased accuracy in modelling could be achieved by combining models. We used a genetic algorithm to ensemble maximally 10 of the top 100 models. The score on the public dataset was used to optimize, with the final ensembled model evaluated on the private dataset. With this method, ensembling achieved a public MCRMSE of 0.2237 (compared to the best public MCRMSE of 0.2276) and a private MCRMSE of 0.3397 (compared to the best private test set MCRMSE of 0.3420). By comparison, averaging the outputs of the top two models gave a result of 0.2244 public, 0.33788 private. Blending the top two solutions with the third solution did not improve the result. An estimated bound of ensembling can be found by optimizing directly to the private ensemble score. With this method, it was possible to achieve a private ensemble score of 0.3382 (again, versus best Leaderboard MCRMSE 0.3420). The improvement of 0.0038 over the leaderboard for this last approach is about the distance between the first-placed and tenth-placed teams, and the ‘correct’ way gives an improvement that is the distance between the first- and fifth-placed teams. All these experiments suggest that most of the signal has been captured by the top two models, and that the use of further ensembling provides, at best, modest improvements. The seemingly puzzling result that the simple ensemble of the top two models outperforms the genetic algorithm blend of the top 10 (on the private test set) suggests that the genetic algorithm did not find a global minimum for model weights.

### Reporting summary

Further information on research design is available in the Nature Portfolio Reporting Summary linked to this article.

## Supplementary information

Supplementary Information Descriptions of model architectures of top 13 models, supplied by Kaggle teams.

Reporting Summary

Supplementary Tables **Supplementary Table 1:** Eterna participants in both ‘Roll-Your-Own-Structure’ labs. **Supplementary Table** **2:** Construct attributes and predictions corresponding to PERSIST-seq independent mRNA degradation test. **Supplementary Table** **3:** List of OpenVaccine Donors.

## Data Availability

All datasets are downloadable in raw RDAT format from https://rmdb.stanford.edu at the following accession numbers: SHAPE_RYOS_0620, RYOS1_NMD_0000, RYOS1_PH10_0000, RYOS1_MGPH_0000, RYOS1_50C_0000, RYOS1_MG50_0000, RYOS2_1M7_0000, RYOS2_MGPH_0000, RYOS2_MG50_0000. Kaggle-formatted train and test sets are downloadable from https://www.kaggle.com/c/stanford-covid-vaccine. Datasets, scripts and models are also included at https://www.github.com/eternagame/KaggleOpenVaccine. Source data are provided with this paper.

## Source data

Source Data Fig. 3 Raw data of public/private MCRMSE scores in Fig. 3a.
Source Data Fig. 5 Raw data of PERSIST-seq half-lives and predictions.
Source Data Extended Data Fig. 1 Raw data of summed degradation rates and degradation rates calculated by dropout rate from reverse transcription.
Source Data Extended Data Fig. 2 Raw data of model scores divided by data type.
Source Data Extended Data Fig. 3 Raw data of RMSE values from Nullrecurrent model by construct.
Source Data Extended Data Fig. 4 Raw data of RMSE values from Nullrecurrent model by construct.

## Code availability

Code to run the Nullrecurrent model and the DegScore-XGBoost model is available at www.github.com/eternagame/KaggleOpenVaccine^36^. Code to use and reproduce the linear regression DegScore model is available at www.github.com/eternagame/DegScore^37^.
