# Supplementary material for: Deep learning models for predicting RNA degradation via dual crowdsourcing
Source: Nat Mach Intell. 2022 Dec 14;4(12):1174–84. doi: 10.1038/s42256-022-00571-8 (PMC9771809; doi:10.1038/s42256-022-00571-8)
Supplement: Supplementary file 2 — Reporting Summary [file 42256_2022_571_MOESM2_ESM.pdf]

nature research

Corresponding author(s): Rhiju Das

Last updated by author(s): 11/30/2022

Reporting Summary

Nature Research wishes to improve the reproducibility of the work that we publish. This form provides structure for consistency and transparency in reporting. For further information on Nature Research policies, see our Editorial Policies and the Editorial Policy Checklist.

Statistics

For all statistical analyses, confirm that the following items are present in the figure legend, table legend, main text, or Methods section.

n/a	Confirmed	
☐	☒	The exact sample size (*n*) for each experimental group/condition, given as a discrete number and unit of measurement
☐	☒	A statement on whether measurements were taken from distinct samples or whether the same sample was measured repeatedly
☐	☒	The statistical test(s) used AND whether they are one- or two-sided<br>*Only common tests should be described solely by name; describe more complex techniques in the Methods section.*
☒	☐	A description of all covariates tested
☐	☒	A description of any assumptions or corrections, such as tests of normality and adjustment for multiple comparisons
☐	☒	A full description of the statistical parameters including central tendency (e.g. means) or other basic estimates (e.g. regression coefficient) AND variation (e.g. standard deviation) or associated estimates of uncertainty (e.g. confidence intervals)
☐	☒	For null hypothesis testing, the test statistic (e.g. *F*, *t*, *r*) with confidence intervals, effect sizes, degrees of freedom and *P* value noted<br>*Give P values as exact values whenever suitable.*
☒	☐	For Bayesian analysis, information on the choice of priors and Markov chain Monte Carlo settings
☒	☐	For hierarchical and complex designs, identification of the appropriate level for tests and full reporting of outcomes
☐	☒	Estimates of effect sizes (e.g. Cohen's *d*, Pearson's *r*), indicating how they were calculated

Our web collection on statistics for biologists contains articles on many of the points above.

Software and code

Policy information about availability of computer code

Data collection	No software was used in data collection.
Data analysis	Mapseeker v2.0 (https://eternagame.org/software) was used to process RNA MAP-seq data for "Roll-your-own-structure" experiments.<br>Custom python scripts to perform data analysis are available at https://www.github.com/eternagame/KaggleOpenVaccine.<br>Python requirements: Arnie (https://github.com/DasLab/Arnie), Python 3.7, numpy 1.19.5, seaborn 0.11.1, scipy 1.3.2.

For manuscripts utilizing custom algorithms or software that are central to the research but not yet described in published literature, software must be made available to editors and reviewers. We strongly encourage code deposition in a community repository (e.g. GitHub). See the Nature Research guidelines for submitting code & software for further information.

Data

Policy information about availability of data

All manuscripts must include a data availability statement. This statement should provide the following information, where applicable:

- Accession codes, unique identifiers, or web links for publicly available datasets
- A list of figures that have associated raw data
- A description of any restrictions on data availability

Data availability. All datasets are downloadable in raw RDAT format from https://rmdb.stanford.edu at the following accession numbers: SHAPE_RYOS_0620, RYOS1_NMD_0000, RYOS1_PH10_0000, RYOS1_MGPH_0000, RYOS1_50C_0000, RYOS1_MG50_0000, RYOS2_1M7_0000, RYOS2_MGPH_0000, RYOS2_MG50_0000. Kaggle-formatted train and test sets are downloadable from https://www.kaggle.com/c/stanford-covid-vaccine.

Field-specific reporting

Please select the one below that is the best fit for your research. If you are not sure, read the appropriate sections before making your selection.

☒ Life sciences ☐ Behavioural & social sciences ☐ Ecological, evolutionary & environmental sciences

For a reference copy of the document with all sections, see nature.com/documents/nr-reporting-summary-flat.pdf

Life sciences study design

All studies must disclose on these points even when the disclosure is negative.

Sample size	Sample sizes for the Kaggle public train set, public test set, and private test set were 2000, 400, and 1801, respectively. No sample size calculations were performed. Sample sizes for high-throughput RNA in-line-seq experiments were determined by the maximum allowable size to achieve sufficient read depth on each library construct.
Data exclusions	Data used for the Kaggle competition training and test set were filtered from the original dataset based on signal-noise ratio and sequence identity, as described in the manuscript. Kaggle competition participants were provided with all of the original dataset.
Replication	1172 of the constructs in the test set were from experiments that had not been finished at the time of the competition end, making the competitor's test set predictions truly blind. The winning models from the Kaggle competition were tested on an independent dataset of mRNA degradation measurements and demonstrated improved performance over simpler biophysics-based models on these predictions as well.
Randomization	Randomization is not relevant because conditions were constructed and there was not subjective allocation of samples to experimental groups.
Blinding	Investigators were not blinded to group allocation as conditions were constructed and there was not subjective allocation of samples to experimental groups.

Reporting for specific materials, systems and methods

We require information from authors about some types of materials, experimental systems and methods used in many studies. Here, indicate whether each material, system or method listed is relevant to your study. If you are not sure if a list item applies to your research, read the appropriate section before selecting a response.

Materials & experimental systems

n/a	Involved in the study
☒	☐ Antibodies
☒	☐ Eukaryotic cell lines
☒	☐ Palaeontology and archaeology
☒	☐ Animals and other organisms
☒	☐ Human research participants
☒	☐ Clinical data
☒	☐ Dual use research of concern

Methods

n/a	Involved in the study
☒	☐ ChIP-seq
☒	☐ Flow cytometry
☒	☐ MRI-based neuroimaging
